# Antiviral strategies against influenza virus: an update on approved and innovative therapeutic approaches

**DOI:** 10.1007/s00018-025-05611-1

**Published:** 2025-02-13

**Authors:** Anna Bonomini, Beatrice Mercorelli, Arianna Loregian

**Affiliations:** 1 Department of Molecular Medicine, University of Padua, Padua, Italy; 2 Microbiology and Virology Unit, Padua University Hospital, Padua, Italy

**Keywords:** Avian influenza, Combination therapy, Protein–protein interaction inhibitors, Hemagglutinin, Neuraminidase, RNA polymerase, Monoclonal antibodies, Targeted protein degradation

## Abstract

Influenza viruses still represent a great concern for Public Health by causing yearly seasonal epidemics and occasionally worldwide pandemics. Moreover, spillover events at the animal-human interface are becoming more frequent nowadays, also involving animal species not previously found as reservoirs. To restrict the effects of influenza virus epidemics, especially in at-risk population, and to prepare a drug arsenal for possible future pandemics, researchers worldwide have been working on the development of antiviral strategies since the 80’s of the last century. One of the main obstacles is the considerable genomic variability of influenza viruses, which constantly poses the issues of drug-resistance emergence and immune evasion. This review summarizes the approved therapeutics for clinical management of influenza, promising new anti-flu compounds and monoclonal antibodies currently undergoing clinical evaluation, and molecules with efficacy against influenza virus in preclinical studies. Moreover, we discuss some innovative anti-influenza therapeutic approaches such as combination therapies and targeted protein degradation. Given the limited number of drugs approved for influenza treatment, there is a still strong need for novel potent anti-influenza drugs endowed with a high barrier to drug resistance and broad-spectrum activity against influenza viruses of animal origin that may be responsible of future large outbreaks and pandemics.

## Introduction

Influenza viruses are viral agents causing high health and economical burdens for human population since they are responsible for annual seasonal epidemics and periodically for pandemics. Influenza A and B viruses (IAV and IBV) are able to infect human airways, causing acute respiratory syndromes characterized by high morbidity and infectivity. These pathogens represent a real threat especially for people at risk of severe complications upon infection, such as immunocompromised patients, elderly, and children [1]. On a global scale, around a billion cases of seasonal influenza (flu) are registered annually, including 3 to 5 milion cases of severe illness, and a number betweeen 290,000 and 650,000 deaths due to respiratory complications (www.who.int). Influenza A viruses, the most common flu agents in human population, are characterized by a considerable evolutionary dynamism given their high mutation rate, which ranges between 10^–3^ and 8 × 10^–3^ substitutions per site per year, resulting in at least one random mutation in each replication cycle [2]. This occurs because the influenza virus RNA-dependent RNA polymerase (RdRp), responsible for both viral transcription and replication, is error-prone due to the low fidelity and absence of proofreading activity. The process of point mutations accumulation during viral genome replication is known as “antigenic drift”. This genomic variability, clustered especially in the exposed portions of viral surface glycoproteins hemagglutinin (HA) and neuraminidase (NA), leads to the generation of new viral variants capable of eluding the host pre-existing immune response, thus eliciting the annual re-emergence of flu seasonal epidemics. Another peculiar feature of influenza viruses is represented by the segmented nature of their genome. This characteristic favours the reassortment of genomic fragments of IAVs of different origin (i.e., human, mammalian, avian) upon co-infection of a permissive host. The reassortment may lead to the generation of a viral progeny antigenically different from the original parental viruses. This process, named “antigenic shift”, is able to generate new IAV strains with pandemic potential [3].

The main strategy for the control of human seasonal epidemics caused by IAV and IBV is vaccination. In fact, anti-influenza vaccination is recommended yearly at the beginning of the cold season for at-risk population for preventing the viral infection [4, 5]. However, anti-flu vaccination presents several drawbacks, mainly including immunological imprinting, limited effectiveness, and the need to reformulate yearly the vaccine due to the high genomic variability of influenza viruses [6, 7]. Despite the annual update, the efficacy of the vaccine can significantly vary because of the possible mismatch between the formulated vaccine components and the actually circulating influenza strains, especially in elderly and immunocompromised individuals [8]. Moreover, in case of the emergence of a new pandemic influenza virus strain, a quite long time would be required for the production of a new vaccine, allowing the rapid spread of the new virus worldwide in the meantime. Given the lack of complete protection offered by the current anti-flu vaccines, in the last 50 years many research efforts have been devoted to the development of anti-influenza drugs for the treatment of viral infections that could be also used as prophylactic agents in at-risk population or in the case of a pandemic emergency. Most of the currently available antivirals are anti-human immunodeficiency virus (HIV) drugs, as since the ‘80s–'90s HIV-associated acquired immunodeficiency syndrome (AIDS) has represented a serious threat to public health and hence both pharmaceutical companies and many academic research groups have focused on the development of new effective anti-HIV agents. Almost all of these compounds were designed to target directly HIV proteins with a pivotal role in the viral replicative cycle, e.g., reverse transcriptase, protease, and integrase [9]. A similar approach has been applied later to develop anti-influenza drugs, focusing mainly on identifying viral targets with an essential function that could be inhibited with small-molecular or peptide-based inhibitors. A feature shared by both HIV and influenza viruses is the high genomic variability, which makes the drug development process even more challenging given the high likeliness of the emergence of resistant viral mutants. To counteract this issue, combination therapies were introduced for the treatment of AIDS, consisting of the simultaneous administration of drugs acting toward different viral targets to obtain a synergistic effect and to reduce the selection of resistant viruses. As described in depth later in this review, this combination strategy was also applied against influenza virus infection in some clinical trials with promising results.

## Clinical management of influenza

**Fig. 1:**
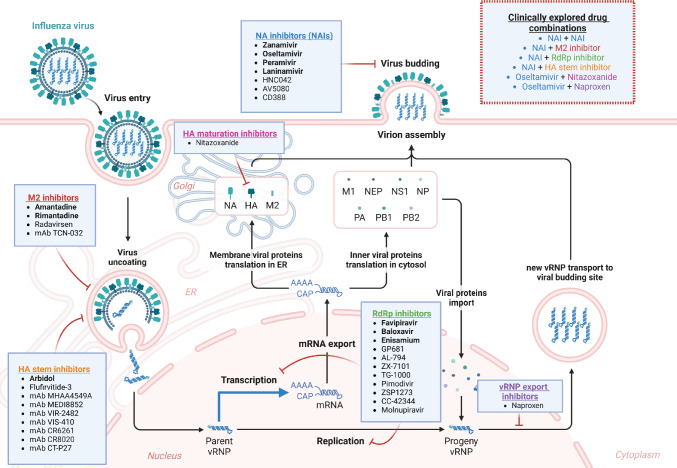
Replication cycle of influenza virus and crucial steps targeted by virus-directed antiviral compounds. Drugs approved for the treatment of influenza virus infection are indicated in bold. In the inset, clinically evaluated drug combinations are reported (Figure created with BioRender)

Currently, anti-flu clinical therapy relies on drugs acting on different steps of the influenza virus replication cycle (Fig. 1); a summary of all the clinically approved anti-influenza agents is reported in Table 1.

**Table 1:** Currently approved drugs for clinical management of influenza

Drug (Trade name)	Viral target	Mode of action	Route of administration	Approval status	References
Amantadine (Symadine®)	IAV M2	Ion channel blocker	Oral	Approved by FDA and EMA (no longer recommended due to resistance)	[12, 345]
Rimantadine (Flumadine®)	IAV M2	Ion channel blocker	Oral	Approved by FDA and EMA (no longer recommended due to resistance)	[12, 345]
Zanamivir (Relenza®)	NA	Sialic acid analogue	Inhalation/intravenous	Approved by FDA and EMA	[16, 17]
Oseltamivir (Tamiflu®)	NA	Sialic acid analogue	Oral	Approved by FDA and EMA	[18, 346]
Peramivir (Rapivab®)	NA	Sialic acid analogue	Intravenous	Approved by FDA and EMA	[19]
Laninamivir (Inavir®)	NA	Sialic acid analogue	Inhalation	Approved only in Japan	[20, 21]
Favipiravir (Avigan®)	RdRp (PB1)	Nucleoside analogue	Oral	Approved only in Japan	[35, 38]
Baloxavir marboxil (Xofluza®)	RdRp (PA)	Cap-dependent endonuclease inhibitor	Oral	Approved by FDA and EMA	[43, 44]
Enisamium Iodide (Amizon®)	RdRp	vRNA synthesis inhibitor	Oral	Approved in selected countries	[48, 49]
Arbidol (Umifenovir®)	HA	Membrane fusion inhibitor	Oral	Approved in Russia and China	[50]

The first anti-influenza drugs, approved in the ‘80 s, were **amantadine** (Symadine®, Symmetrel®) and **rimantadine** (Flumadine®), inhibitors of the viral ion channel M2 that is responsible for the late endosomes acidification and subsequent viral uncoating [10]. In detail, these drugs bind to the inner surface of M2, thus stabilizing it in a closed conformation and halting proton flux through the channel [11]. Amantadine and rimantadine are administered orally and present efficacy only against IAV due to the amino acid sequence diversity of IBV M2 protein [10]. Nowadays, these adamantanes are no longer administered in clinics because of the massive and rapid emergence of drug-resistant influenza strains, in addition to the severe side effects [12, 13]. The main mutations conferring resistance to adamantanes affect residues located in the transmembrane domain of M2 (i.e., L26, V27, A30, S31, G34, and L38), which interact with the drugs in the inner ion channel. Among these mutations, the most frequent is the S31N substitution, found in almost 95% of resistant viruses, along with the substitution V27A, detected in about 1% of resistant strains. These amino acid substitutions have been demonstrated not to impair viral replicative fitness, virulence, or transmissibility, thus allowing the rapid spread of these viral variants worldwide up to > 99% prevalence of currently circulating strains [14].

**Fig. 2:**
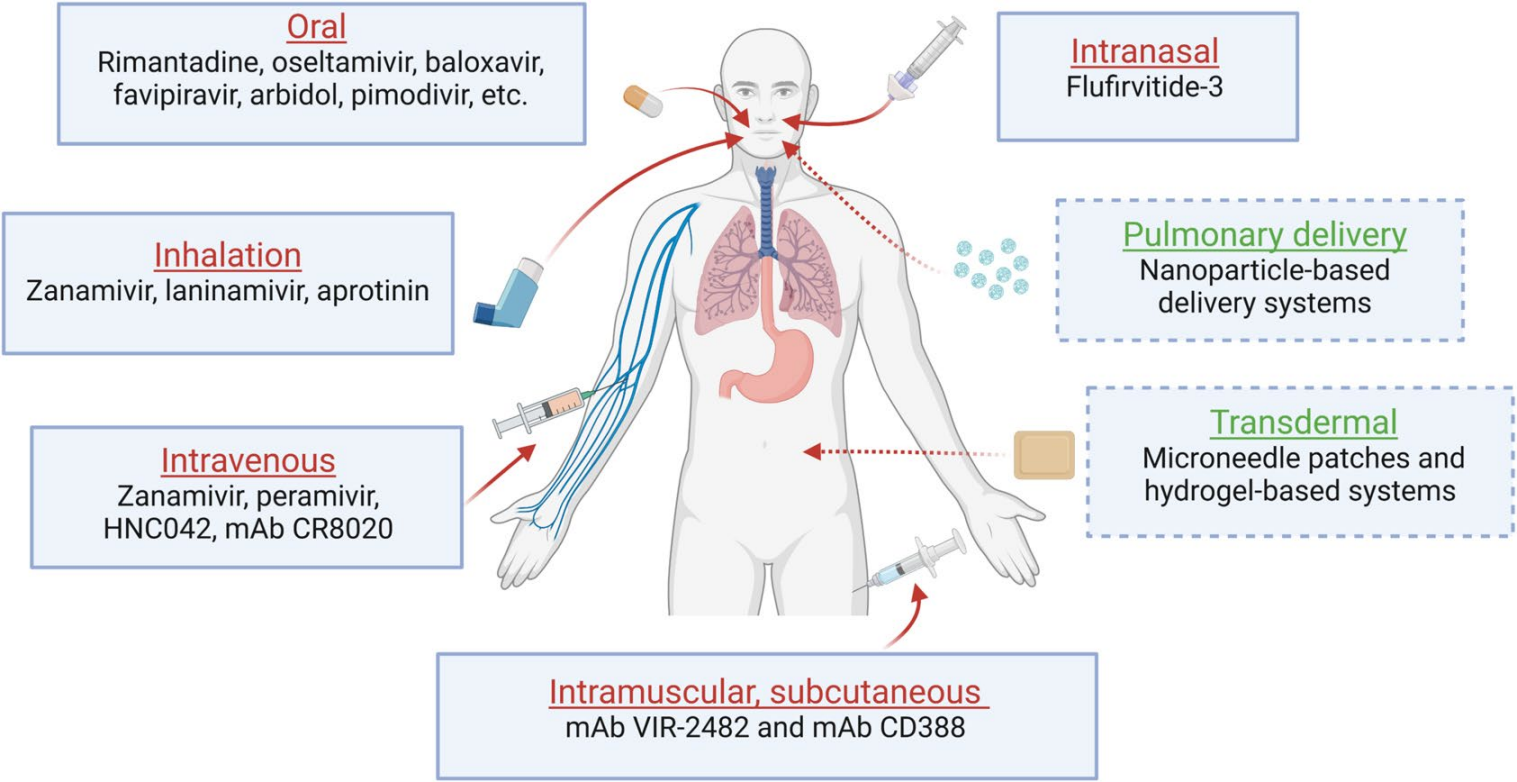
Administration routes of anti-flu agents. Well-established routes of administration of anti-influenza compounds and mAbs are shown in red. Novel alternative strategies of administration recently investigated are also depicted (in green). (Figure created with BioRender)

The second class of anti-influenza agents authorized for clinical therapy is represented by the NA inhibitors (NAIs), able to prevent the cellular sialic acid (SA) cleavage by viral NA, which is fundamental for the efficient detachment and release of the newly synthesized virions from the infected host cell (Fig. 1). Moreover, the cleavage of host SA, one of the main constituents of respiratory airway mucus, is highly important for allowing the initial penetration of the virus into the respiratory epithelium. Most of these NAIs have been designed as structural analogues of SA, to produce molecules endowed with high binding affinity and competing with the natural substrate for the NA catalytic site. These drugs are active against both IAV and IBV, as the active site of viral NA is highly conserved among all influenza types [15]. NAIs should be administered to the patients within 48 h from the symptoms onset to result effective. The first drug approved as NAI was **zanamivir** (Relenza®), which was licensed in 1999. This drug is a synthetic analogue of SA and is administered by inhalation, due to its low oral bioavailability. It is employed for the treatment of uncomplicated acute infections by influenza viruses and is recommended also for the prophylaxis of flu in adults and children over 7 years old [16]. Besides the standard administration route by inhalation, in 2019 an intravenous zanamivir formulation (Dectova®) was approved by the European Medicines Agency (EMA) for use in hospitalized and critically ill patients (Fig. 2), who are unable to assume antiviral drugs orally or by inhalation, and for use under exceptional circumstances (e.g., in case of resistance to other anti-influenza drugs) [17].

Currently, the Standard-of-Care (SOC) for influenza virus infection prophylaxis and treatment is **oseltamivir** (Tamiflu®), which was approved by main regulatory agencies after zanamivir in 1999. This drug is administered as a prodrug, oseltamivir phosphate, which is then hydrolysed by hepatic esterases into its active form, oseltamivir carboxylate, an analogue of SA as zanamivir. This NAI is administered orally (Fig. 2) and is employed for the treatment of uncomplicated acute influenza virus infections in adults and infants over 2 weeks of age, and for flu chemoprophylaxis in adults and children over 1 year old [18]. More recently, in 2014, a further drug was approved by the main regulatory agencies—**peramivir** (Rapivab®)—a novel cyclopentane neuraminidase inhibitor. This drug is administered intravenously (Fig. 2) for the treatment of uncomplicated acute flu in patients over 2 years old within 2 days from the onset of influenza-related symptoms [19]. Furthermore, there is another promising synthetic analogue of SA still under clinical investigation, **laninamivir** (Inavir®), which has received so far conditional approval only in Japan. This drug is administered by inhalation (Fig. 2) as a prodrug, laninamivir octanoate, which is then converted into its corresponding active form in the respiratory tract. This NAI acts against both IAV and IBV, including oseltamivir-resistant viral strains. To date, laninamivir exhibited in the clinical trials the ability to reduce the number of infected patients spreading virus [20] and to prevent the emergence of clinical flu symptoms when used as a post-exposure prophylactic measure [21].

Despite the high conservation of the NA catalytic site, some viral variants with reduced susceptibility to NAIs have emerged. This lower sensitivity to NA inhibitors can be conferred by the substitution of the following residues (all localized close to the active site): SE105, E119, I122, Q136, D151, R152, D198, R224, S246, H275, R292, N294, and R371. The most common NA mutation is the H275Y substitution (H275 and H274 in N1 and N2 numbering, respectively), which confers resistance to both oseltamivir and peramivir, but not to zanamivir and laninamivir [22, 23]. In contrast to the mutations conferring resistance to adamantanes, this amino acid substitution proved to significantly affect viral fitness both in vitro and in vivo*,* by impairing both viral replication and transmission [24, 25]. Nevertheless, this mutation presented a very high prevalence in A(H1N1) influenza viruses circulating in the late 2000s, even reaching 100% prevalence in Japan in the 2008–2009 season [26]. The rapid and wide spread of these biologically disadvantaged viruses was made possible by the simultaneous emergence of permissive mutations, in particular the D344N substitution, which compensated for the fitness loss due to the H275Y change by significantly enhancing NA catalytic activity [27]. These A(H1N1) oseltamivir-resistant viruses were afterward completely replaced by the emergence of the pandemic strain A(H1N1)pdm09 in 2009. Compared to the previously circulating A(H1N1) strains, this strain did not harbour the H275Y substitution in NA and resulted less prone to acquire both this and other substitutions. This led to a substantial decrease in oseltamivir-resistant strains circulating worldwide, reaching nowadays a prevalence lower than 1% [28]. Since 2009, some community clusters of oseltamivir-resistant A(H1N1)pdm09 viruses have been identified in some countries worldwide (especially in America and Asia). These resistant variants were isolated mainly in immunocompromised patients receiving oseltamivir therapy, as a result of selective drug’s pressure [29]. By sequencing these resistant strains, it was discovered that they possessed some complementary substitutions, i.e., V241I, N369K, N386K, and N200S, able to compensate for the detrimental effects of the H275Y change [30–33]. Hence, monitoring of circulating influenza strains is crucial to timely identify oseltamivir-resistant strains and to prevent a possible sudden spread of drug resistance.

A further category of available anti-flu therapeutics is represented by the viral RNA polymerase inhibitors. The influenza virus RdRp is responsible for both viral transcription and replication and is a highly conserved heterotrimeric complex composed by three subunits, named PA, PB1, and PB2 [34]. **Favipiravir** (Avigan®) is a nucleoside analogue acting as an inhibitor of PB1, which is the subunit of the RdRp complex responsible for the synthesis of new viral RNA in both transcription and replication processes. To date, this drug has received only limited conditional approval in Japan for the oral treatment of severe influenza virus infection [35]. Favipiravir is a purine nucleoside phosphorylated by cellular kinases into ribofuranosyl triphosphate, which is then inserted into the nascent viral RNA chain, causing the insertion of multiple lethal mutations [36] (Fig. 1). More rarely, it also acts as a chain terminator, mainly when two favipiravir molecules are incorporated consecutively [37]. It showed antiviral activity against influenza A, B, and C viruses, and also against other RNA viruses, including flaviviruses, arenaviruses, bunyaviruses, noroviruses, alphaviruses, and coronaviruses [38]. During the recent SARS-CoV-2 pandemic, this drug was approved for emergency use for treating COVID-19 (www.fda.gov; www.ema.europa.eu). Unfortunately, it is associated to serious side effects, including teratogenicity and embryotoxicity, thus it is not approved for use during pregnancy [39]. Although an in vitro study reported the emergence of favipiravir-resistant influenza viruses, to date clinically isolated viral strains with mutations conferring resistance to favipiravir have not been reported yet [40, 41].

More recently, a new class of RdRp inhibitors targeting the N-terminal endonuclease domain of the PA subunit has been developed. These molecules are cap-dependent endonuclease inhibitors (CENI). The PA endonuclease plays a pivotal role in the “cap-snatching” process, a crucial step in the initiation of viral transcription. This process consists first in the 5′-cap binding by the PB2 subunit and then in the cleavage of nascent cellular pre-mRNAs by the PA subunit. The cap-snatching generates short 5′-capped RNA oligos that are used by the PB1 subunit as primers for starting viral transcription [42]. So far, one CENI, i.e., **baloxavir marboxil** (Xofluza®, Fig. 1), was approved in 2018 by FDA and in 2021 by EMA for the treatment of uncomplicated influenza virus infections by IAV and IBV [43]. Since 2020 it has been also authorised as a post-exposure intervention, in adults and children aged 5 years and older [44]. Baloxavir is administered orally (Fig. 2) as a prodrug, baloxavir marboxil (BXM), which is then metabolized into its active form, baloxavir acid (BXA). Unfortunately, as well as adamantanes and NAIs, BXM can also induce the emergence of drug resistance, mainly due to substitutions of the residue I38, which is crucial for the correct positioning of BXA into the active site of PA endonuclease. It was demonstrated that substitutions of the I38 residue severely impair PA endonuclease activity, thus decreasing significantly viral replicative fitness [45, 46]. PA I38X variant viruses were almost exclusively isolated from infected patients after exposure to the drug, as early as 24 h post-treatment initiation [45, 47]. Hence, baloxavir resistance does not currently represent a great concern, since it is induced by the selective pressure of the administered drug, which so far has been limitedly used for influenza treatment worldwide.

Another marketed drug targeting the viral RNA polymerase is **Enisamium iodide** (Amizon®), an isonicotinic acid derivative currently licensed in the Commonwealth of Independent States, in former Soviet Union countries, and in Mongolia as an anti-influenza agent. This compound acts by inhibiting both IAV and IBV RNA synthesis via its hydroxylated metabolite, VR17-04 [48] (Fig. 1), but its exact mechanism still needs to be further investigated. Enisamium iodide recently underwent a phase II clinical trial (NCT04682444) in patients with confirmed influenza in whom it demonstrated a reduction of viral shedding and of the duration of influenza-related symptoms compared to placebo [49].

A further commercialized drug against influenza viruses is **arbidol** (Umifenovir®), which to date has received limited authorization in Russia and China for flu prophylaxis and treatment. This compound is an indole derivative able to impair IAV and IBV HA-induced membrane fusion by binding to the hydrophobic cavity of HA stalk, thus preventing the introduction of its fusion peptide into the endosomal membrane [50] (Fig. 1). This drug results mostly effective in the acute phase of the influenza disease. As favipiravir, arbidol is endowed with antiviral activity against a broad variety of viruses including SARS-CoV-2, Lassa virus, Ebola virus, flaviviruses, etc. [51–53].

## Anti-flu therapeutics under clinical development

**Fig. 3:**
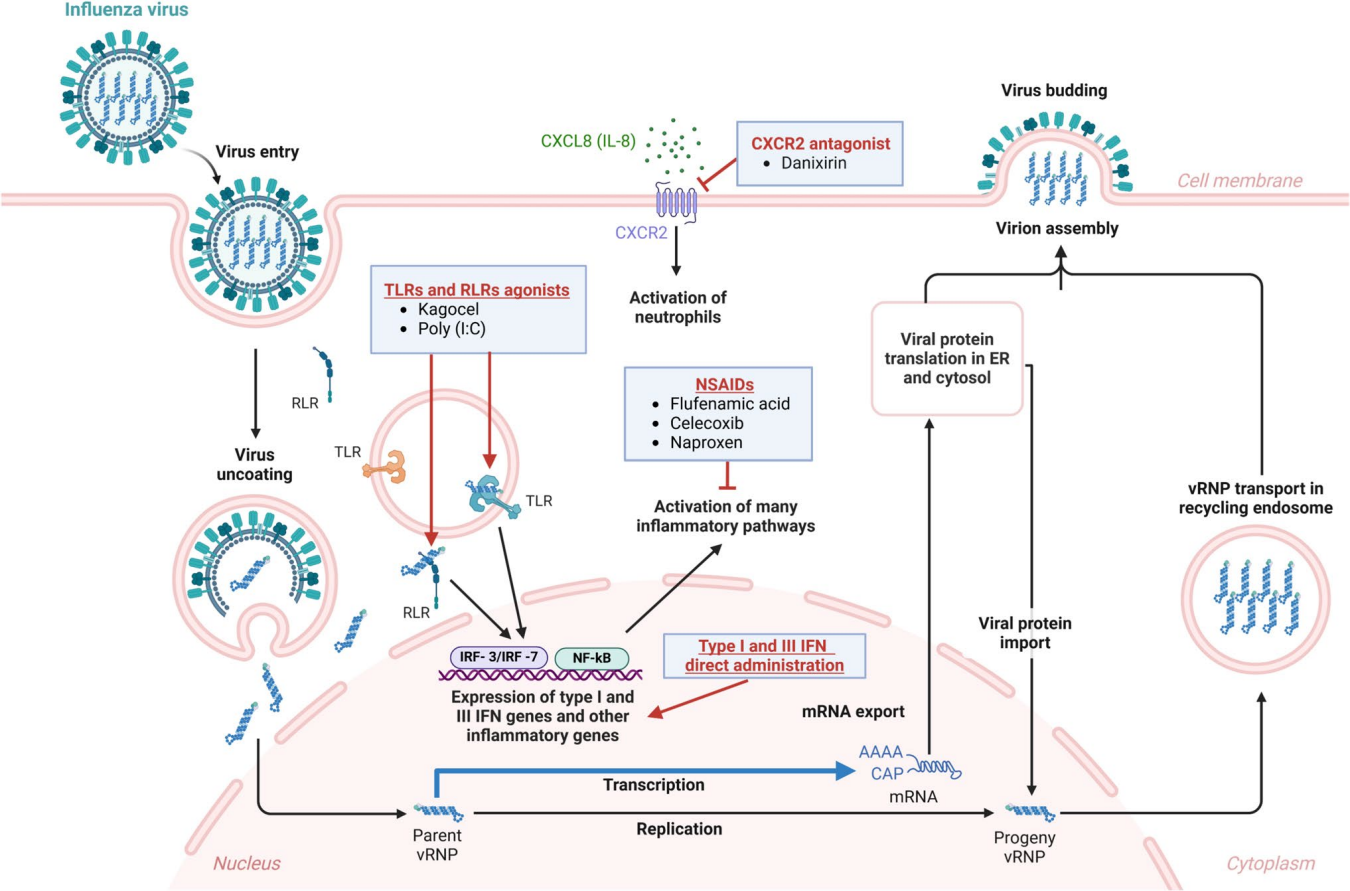
Immunomodulatory and anti-inflammatory agents for the treatment of influenza virus infection. The four main classes of host-targeted immunomodulatory and anti-inflammatory agents repurposed for flu therapy are shown, along with the molecular targets and cellular pathways that they modulate in the context of host cell immune response. *IFN* interferon, *NSAIDs* nonsteroidal anti-inflammatory drugs, *RLRs* Rig-I-like receptors, *TLRs* Toll-like receptors. (Figure created with BioRender)

To date, several anti-influenza therapeutics are under clinical evaluation. These can be divided mainly into two groups based on the molecular target: (i) virus-directed drugs that act toward a viral target at a certain step of replication cycle (Fig. 1), and (ii) host-directed drugs that target host cell factors involved in influenza virus replication and pathogenesis (Fig. 3).

### Virus-directed drugs

The majority of anti-flu therapeutics under clinical investigation are virus-directed agents. Starting from the entry phase, some monoclonal antibodies (mAbs) have been developed that bind to the viral hemagglutinin (HA). These mAbs target either the HA globular head, to prevent its binding to the host SA receptor, or the HA stalk, to impair fusion between the viral envelope and the cell membrane (Table 2). Since the HA stem is the most conserved part of this viral glycoprotein, antibodies directed toward this region are typically able to broadly neutralize multiple IAV and IBV subtypes. Differently, the HA head is characterized by high sequence variability and various glycosylation signatures among the different influenza subtypes [54]. For this reason, to date only mAbs targeting HA stalk have achieved clinical evaluation, but none of them has entered Phase III yet.

**Table 2:** Virus-directed anti-influenza agents under clinical and preclinical development

Target	Agent		Mode of action	Stage	References
HA	mAbs	CR8033 and C12G6	Viral entry inhibitors (targeting HA head RBD) + Fc-mediated immune functions	Preclinical, in vivo (mice)	[149]
		C05		Preclinical, in vivo (mice)	[144]
		S139/1		Preclinical, in vitro	[145]
		F045-092		Preclinical, in vitro	[146]
		S5V2-29 and H2214		Preclinical, in vivo (mice)	[148]
		Flu-20		Preclinical, in vivo (mice)	[147]
		CR6261 and CR8020	Membrane fusion inhibitors (targeting HA stem) + Fc-mediated immune functions	Clinical, phase II (CR6261: NCT02371668; CR8020: NCT01938352)	[56–58]
		MHAA4549A		Clinical, phase II (NCT01980966; NCT02293863; NCT02623322)	[60–62]
		MEDI8852		Clinical, phase II (NCT02603952)	[64, 65]
		VIR-2482		Clinical, phase I (NCT04033406)	[63, 66]
		VIS-410		Clinical, phase II (NCT02989194; NCT02468115)	[67–69]
		CT-P27		Clinical, phase II (NCT02071914; NCT03511066)	[71]
		31.a.83 and 56.a.09		Preclinical, in vitro	[154]
		FI6v3		Preclinical, in vivo (mice)	[153]
		CR9114		Preclinical, in vivo (mice)	[149]
	Synthetic compounds	JNJ4796		Preclinical, in vivo (mice)	[161]
		ING-4666		Preclinical, in vivo (mice)	[162, 163]
		Nitazoxanide	HA maturation inhibitor	Clinical, phase IIb/III (NCT01227421)	[104, 105]
M1/M2	Oligonucleotide	Radavirsen (AVI-7100)	Antisense oligonucleotide targeting M1 and M2	Clinical, phase I (NCT01747148)	[73]
M2	mAb	TCN-032	Viral budding inhibitor + Fc-mediated immune functions	Clinical, phase II (NCT01719874)	[74, 75]
NP	Synthetic compounds	Naproxen	vRNP nuclear export inhibitors	Clinical, observational study (NCT04315194)	[95, 96]
		ZBMD-1		Preclinical, in vivo (mice)	[228]
		KR-23502		Preclinical, in vitro	[227]
		FA-6005		Preclinical, in vitro	[229]
		Nucleozin	NP oligomerization inhibitor	Preclinical, in vivo (mice)	[226]
		KR424	NP/viral RNA interaction inhibitors	Preclinical, in vivo (mice)	[221]
	Natural compound	Mycalamide A		Preclinical, in vitro	[222]
PB2	Synthetic compounds	Pimodivir	PB2 cap-binding domain (CBD) inhibitors	Clinical, phase II (NCT02532283)	[77, 141]
		ZSP1273		Clinical, phase III (NCT04683406)	[78, 80]
		CC-42344		Clinical, phase II (NCT06160531)	[81]
		Cap-3 and Cap-7		Preclinical, in vitro	[248]
		PB2-39		Preclinical, in vivo (mice)	[250]
	Natural compound	Dihydromyricetin		Preclinical, in vitro	[249]
PA	Synthetic compounds	ZX-7101	Cap-dependent endonuclease inhibitors (CENI)	Clinical, phase I (NCT05217732)	[82]
		GP681		Clinical, phase III (NCT05474755)	-
		AL-794		Clinical, phase I (NCT02877160; NCT02588521)	[84, 85]
		TG-1000		Clinical, phase II (NCT04706468)	[86]
		R05-3A and R05-2		Preclinical, in vitro	[236]
		Compounds 14 and 19		Preclinical, in vitro	[242]
	Natural compounds	Marchantins A and E		Preclinical, in vitro	[244]
	Synthetic compound	Compound 54	PA/PB1 interaction inhibitor	Preclinical, in ovo	[273, 282]
PB1	Synthetic compounds	Molnupiravir	Nucleoside analogue	Clinical, phase II (NCT05818124)	[93]
		Compound 367	Non-nucleoside PB1 inhibitors	Preclinical, in vitro	[223]
		ASN2		Preclinical, in vivo (mice)	[251]
		11q		Preclinical, in vivo (mice)	[252]
NA	Synthetic compounds	HNC042	Sialic acid analogues	Clinical, phase I (NCT04603989)	-
		AV5080		Clinical, phase III (NCT05093998)	[107]
	Conjugate of a NAI + mAb	CD388	Sialic acid analogue + Fc-mediated immune functions	Clinical, phase II (NCT05523089)	[108]
	Synthetic compound	2,3-difluorosialic acid	Covalent NA catalytic inhibitor	Preclinical, in vivo (mice)	[206]
	mAbs	IG01	Steric interference with NA’s access to SA + Fc-mediated immune functions	Preclinical, in vivo (mice)	[216]
		DA03E17		Preclinical, in vivo (mice)	[217]
		1G05 and 2E01		Preclinical, in vivo (mice)	[218]
		FNI9		Preclinical, in vivo (mice)	[219]
NS1	Natural compounds	Epigallocatechin gallate (EGCG)	NS1/viral dsRNA interaction inhibitor	Preclinical, in vitro	[318]
		Baicalin	NS1/p85β interaction inhibitor	Preclinical, in vivo (mice)	[322]
	Synthetic compounds	A9 and A22	NS1/CPSF30 interaction inhibitor	Preclinical, in vitro	[319]

The first anti-HA mAbs showing promising results in preclinical studies were **CR6261** and **CR8020**, human antibodies generated by phage display technology. The first binds to the conserved hydrophobic pocket on HA stalk, whereas the latter stabilizes the structure of the fusion peptide, thus preventing low pH-mediated HA conformational changes and subsequent membranes fusion process [55] (Fig. 1). In vitro, CR6261 exhibited broad-spectrum neutralizing activity against H1, H2, H5, H6, H8, H9, H13, and H16 subtypes [56, 57], while CR8020 neutralized only the H3, H7, and H10 subtypes [57]. In vivo, CR6261 demonstrated efficacy against H5N1 and H1N1 infection in mice as both prophylactic and therapeutic treatment [56], while CR8020 proved to effectively prevent and protect mice from infection with lethal doses of H3N2 and H7N7 [57]. Despite the promising preclinical results, in a subsequent phase II trial (NCT02371668) CR6261 failed to demonstrate protective or therapeutic effect in healthy volunteers challenged with an influenza H1N1 virus [58]. About 10 years ago, a phase I (NCT01756950) and a phase II clinical trial (NCT01938352) evaluating safety and efficacy of CR8020 intravenous administration, respectively, were completed, but the results were never posted.

Another HA stem-targeting antibody that has achieved clinical investigation is **MHAA4549A**, a human IgG1 mAb that targets the top of the highly conserved helix of HA stalk and is able to neutralize a broad variety of IAVs [59]. MHAA4549A demonstrated to be well tolerated and significantly decreased virus shedding and influenza-related symptoms in a human challenge phase IIa clinical trial (NCT01980966) [60]. Nonetheless, this neutralizing mAb did not result clinically effective in both a phase IIb study (NCT02293863), combining MHAA4549A and oseltamivir in patients hospitalized with severe influenza [61], and another phase II trial (NCT02623322) in patients with acute uncomplicated IAV infection [62]. A further human neutralizing mAb under clinical evaluation for flu therapy is **MEDI8852**. This molecule binds to the hydrophobic groove of HA stem, like CR6261, and to most of its fusion peptides [63]. MEDI8852 exhibited promising results in preclinical studies, especially in vivo demonstrating a more potent therapeutic effect compared to oseltamivir [64]. Despite these promising results, in a phase IIa clinical trial (NCT02603952) MEDI8852 treatment resulted in slightly more adverse effects than the oseltamivir monotherapy and did not provide any significant clinical advantage over the SOC therapy, neither in terms of viral load reduction nor in flu-related symptoms alleviation. Hence, the producing company decided to interrupt the clinical trial [65]. Successively, starting from MEDI8852, a new anti-HA stem mAb was developed, **VIR-2482** [63]. This antibody was synthesized by inserting into the Fc region of MEDI8852 the “LS mutation” (M428L/N434S) to prolong elimination half-life via increased neonatal Fc receptor (FcRn)-mediated antibody recirculation. In a phase I study in healthy volunteers (NCT04033406), VIR-2482 exhibited a good safety, tolerability, and pharmacokinetic profile [66]. However, a subsequent phase II trial (NCT05567783) with this mAb was terminated ahead of time since intramuscular administration of VIR-2482 failed in achieving its primary efficacy endpoint in the prevention of seasonal flu illness.

The human-engineered mAb **VIS-410** targets unique epitopes on the HA stalk region and is endowed with broad neutralizing activity against multiple strains of IAV. Notably, VIS-410 showed protective effects in mice against lethal challenge with A(H7N9) influenza virus [67]. In a phase II clinical trial (NCT02989194), VIS-410 administration to patients with complicated IAV infection was associated with a significant decrease of late-infection viral loads and of time to virus clearance [68]. Moreover, this mAb demonstrated clinical efficacy in reducing viral shedding in another phase II study (NCT02468115) in which healthy volunteers were challenged with an IAV H1N1 strain [69].

Unfortunately, like small-molecule antivirals, mAbs treatment can also select for resistant mutants. In addition, circulating influenza viruses may already carry mutations that significantly reduce viral susceptibility to mAbs [54]. Moreover, it was observed that H3 subtype viruses are endowed with a significantly lower genetic barrier to resistance to HA-targeted mAbs than H1 influenza strains. This diversity is related to structural differences in HA stem between the two subtypes [70]. In order to limit viral mutants escape, epitopes closer to the viral envelope should be preferred given their higher conservation level. Another possible strategy consists of the simultaneous administration of two or more antibodies targeting different viral epitopes, which can act synergistically, thus significantly enhancing the antiviral efficacy of the treatment. This combination approach was recently applied leading to the development of **CT-P27**, a novel cocktail of anti-HA antibodies. CT-P27 is a mixture of CT149 and CT120, two human mAbs targeting epitopes on HA stem very close to each other but without generating any mutual interference. This combination of antibodies revealed in vitro broad-spectrum neutralizing activity against IAV and demonstrated in vivo both therapeutic and prophylactic efficacy, as well as synergistic effect with oseltamivir in influenza virus-challenged mouse models [71]. Two phase II clinical trials to evaluate CT-P27 safety and efficacy were completed. In NCT02071914, CT-P27 administration to healthy patients challenged with an IAV strain resulted in a reduction of viral load compared to the placebo. In NCT03511066, patients with acute uncomplicated influenza demonstrated a shortening of time to resolution of flu symptoms upon CT-P27 infusion compared to placebo.

In addition to HA stalk-directed mAbs, another antiviral agent acting as an inhibitor of influenza virus entry currently under clinical investigation is **Flufirvitide 3** (Fig. 1). This drug is a 16-aminoacid peptide derived from a specific HA2 region named the fusion initiation region. This peptide possesses in vitro broad-spectrum inhibitory effect against both IAV and IBV [72]. To date, two phase I clinical trials have been performed to evaluate the safety of Flufirvitide 3 nasal spray formulation in healthy subjects (NCT01313962) and to assess the safety, tolerability, and pharmacokinetic profile of the dry powder form of the peptide (NCT01990846). However, the results of both these clinical trials are not yet available.

To induce fusion peptide insertion into the endosomal membrane and the release of bound viral ribonucleoprotein (vRNP) by viral matrix protein M1, a decrease of the pH in the late endosomes is required. This reduction is mainly driven by the viral ion channel M2, which is thus an attractive target for anti-influenza drugs design. Besides the previously reported adamantanes, currently two novel ion channel inhibitors are being evaluated in clinical settings, i.e. **radavirsen (AVI-7100)** and **TCN-032** (Fig. 1 and Table 2). Radavirsen is a phosphorodiamidate morpholino oligomer that targets the initiation start site of M1 and M2 mRNA transcripts, thereby preventing their translation. Radavirsen has demonstrated a good safety profile in a phase I clinical trial (NCT01747148), but more studies will be required to evaluate its therapeutic potential against the influenza virus [73]. TCN-032 is a human monoclonal antibody targeting the M2 extracellular region (M2e), which is particularly conserved among IAVs. Differently from HA stalk-directed mAbs, TCN-032 is devoid of neutralizing activity, but it blocks new influenza virions assembly by hampering viral budding, thus limiting viral spreading. Moreover, this mAb exerts its antiviral effect by binding directly M2e protein present on free virus, thereby triggering clearance mechanisms, e.g., viral aggregation, opsonophagocytosis, or antibody-dependent virolysis [74]. In a phase II clinical study (NCT01719874) in healthy people challenged with a H3N2 strain, TCN-032 demonstrated a good tolerability and pharmacokinetic profile, without showing evident adverse effects, but it did not significantly ameliorate flu-related symptoms [75].

In addition to the already marketed baloxavir marboxil and favipiravir, other drugs directed against the influenza virus RdRP are currently under clinical investigation. Among these antiviral agents, a novel class of RdRp inhibitors targeting the viral PB2 subunit is emerging [76]. One of the first developed anti-PB2 compounds was **pimodivir** (VX-787; JNJ-63623872) (Fig. 1 and Table 2). It is a non-nucleoside inhibitor capable of occupying the central cap-binding domain (CBD) of PB2, thereby preventing its binding to the 5′-cap of cellular pre-mRNAs. Despite some encouraging initial results in the clinical trials [77], in 2020 clinical developmental program for pimodivir was discontinued by Janssen, since the drug did not demonstrate any benefit over SOC in infected patients (www.janssen.com/clinical-trials). Nevertheless, this drug is still being evaluated in clinical trials in combination with other anti-flu agents to assess any possible synergistic activity. Currently, two other PB2 CBD inhibitors are undergoing clinical assessment, **ZSP1273** and **CC-42344** (Table 2). ZSP1273 is a small molecule able to inhibit in vitro both H1N1 and H3N2 IAVs [78]. This compound underwent a phase I clinical trial (NCT03679143) in which it showed favourable safety, tolerability, and pharmacokinetic profiles [79]. Moreover, ZSP1273 proved to be clinically effective in reducing flu-related symptoms and in decreasing viral load in adult subjects with acute uncomplicated influenza infection in a phase II trial (NCT04024137) [80]. Recently, a phase III clinical study aimed at comparing the antiviral efficacy of ZSP1273 to placebo or oseltamivir in patients with uncomplicated influenza was completed (NCT04683406), but results have not been posted yet. CC-42344 is another small-molecule inhibitor of PB2 that provided promising results in vitro exhibiting antiviral activity in the nanomolar range against a panel of seasonal and pandemic IAV strains [81]. In 2023, a phase I clinical trial testing its safety, tolerability, and pharmacokinetics was concluded (NCT05202379), but the results are still not available. In the meantime, recruitment for a viral challenge study with CC-42344 in healthy volunteers has been already commenced (NCT06160531).

Besides baloxavir marboxil (BXM), other CENI were developed and reached clinical evaluation (Fig. 1). Compound **ZX-7101** was derived from BXM and is a prodrug that is metabolized in vivo into its active form, ZX-7101A. This compound resulted active in vitro against a wide panel of IAV subtypes, with activity in the nanomolar range comparable to BXA. Moreover, administration of ZX-7101 to mice challenged with a lethal H1N1 virus exerted a significant protective effect, resulting in reduction of viral RNA loads and pulmonary damage [82]. Recently, a phase I study to assess safety, tolerability, and pharmacokinetics of ZX-7101 was completed (NCT05217732). Another cap-snatching inhibitor under clinical investigation is **GP681**, a prodrug converted in vivo into its active form, the metabolite GP1707007. This compound was evaluated for its safety and antiviral efficacy in subsequent phase I, II, and III clinical trials (NCT04729764; NCT04736758; NCT05474755), but the results of these studies have not been posted yet. A further orally active inhibitor of PA endonuclease under clinical evaluation is **AL-794**, prodrug of ALS-033719, which revealed in vitro antiviral efficacy both against IAV and IBV [83]. This prodrug underwent two phase I clinical studies. In the first (NCT02877160), AL-794 revealed a good safety, tolerability, and pharmacokinetic profile in healthy individuals [84], while in the subsequent one (NCT02588521), a challenge study in healthy subjects inoculated intranasally with an IAV H3N2 strain, a reduction of flu-associated symptoms, viral loads, and mucus weight was associated with AL-794 administration [85]. Unfortunately, after these studies development of AL-794 was discontinued because clinicians were not able to establish an effective and well-tolerated drug dosage to move forward to phase II. The company Tiagen Biotechnology in Taiwan developed **TG-1000**, a novel CENI, prodrug of TG-0527 that exhibited inhibitory activity against IAV, IBV, and oseltamivir-resistant strains in vitro. In a phase I clinical trial (NCT04495322), TG-1000 showed a good safety, tolerability, and pharmacokinetic profile in healthy volunteers [86]. Recently, a phase II study (NCT04706468) to assess the antiviral efficacy and safety of TG-1000 in adult patients with uncomplicated influenza was completed.

In addition to favipiravir, another ribonucleoside analogue was identified as a potential anti-flu drug, i.e., **molnupiravir** (**EIDD-2801)** (Fig. 1). This molecule is a prodrug of N4-hydroxycytidine (NHC), a pyrimidine analogue with broad-spectrum antiviral activity against several viral families, including flaviviruses, alphaviruses, coronaviruses, and togaviruses [87–91]. Molnupiravir (Lagevrio®) was approved by main regulatory agencies in 2021 during SARS-CoV-2 pandemic for emergency use for treating mild-moderate COVID-19. This compound acts akin to favipiravir by introducing frequent transition mutations, thereby eliciting an error catastrophe during both viral transcription and replication [92]. In vitro, this molecule proved to impair both IAV and IBV replication in human airway epithelium model cultures. In ferrets infected with H1N1 and H3N2 strains, oral administration of molnupiravir reduced significantly viral load in both the upper and lower respiratory tract and flu-related symptoms [93]. Similarly to favipiravir, molnupiravir has so far revealed a high barrier to drug-resistance, as many preclinical studies have unsuccessfully attempted to induce the emergence of viral escape mutants. The major concern with this drug is its potential cytotoxicity due to incorporation of this nucleoside analogue also into host RNAs by cellular RNA polymerases, in particular by mitochondrial RNA polymerases, which are devoid of proofreading activity. A phase II clinical trial (NCT05818124) to evaluate safety and efficacy of molnupiravir in healthy patients challenged with an IAV H1N1 strain was recently completed, but the results have not been published yet.

Besides targeting the subunits of the RdRp, it is also possible to address the viral nucleoprotein (NP), which has a pivotal role in the assembly of vRNP complexes and also in their transport between cellular cytoplasm and nucleus [94]. **Naproxen**, a non-steroidal anti-inflammatory drug, is currently under clinical investigation as an anti-flu agent given its antiviral effect on NP. In fact, this agent showed ability to impair IAV and IBV replication both in vitro and in vivo through the disruption of the interaction between NP and the host protein CRM1 that mediates the export of viral vRNPs (Fig. 1) [95]. An observational clinical study (NCT04315194) revealed that the administration of combined therapy with naproxen, clarithromycin, and oseltamivir in hospitalized adult patients with H3N2 infection reduced mortality and hospitalization duration compared to the control group treated with oseltamivir monotherapy [96].

Another example of repurposed drug against influenza virus currently under clinical evaluation is represented by **nitazoxanide**. This drug is approved by the main regulatory agencies as an anti-protozoan agent and also demonstrated some efficacy against intestinal parasites like tapeworms and helminths by impairing mitochondrial ATP production [97]. Furthermore, nitazoxanide also exhibited broad-spectrum antiviral activity against influenza viruses, MERS-CoV, SARS-CoV-2, norovirus, and paramyxovirus [98–101]. Nitazoxanide exerts its anti-influenza virus effect by hampering HA transport from the endoplasmic reticulum to the Golgi complex for HA terminal glycosylation, thereby preventing hemagglutinin maturation and subsequent correct assembly of the new influenza virions (Fig. 1) [102]. Nitazoxanide exhibited in vitro potent inhibitory activity against IAV H1N1 and H3N2 and against IBV [103], and in vivo*,* upon combination with oseltamivir, it showed higher antiviral efficacy in preventing H1N1 infection compared to oseltamivir monotherapy [104]. A phase IIb/III trial (NCT01227421) revealed that treatment for 5 days with nitazoxanide in patients with acute uncomplicated influenza shortened flu symptoms duration [105]. In contrast, a more recent phase II clinical trial (NCT02057757) failed in demonstrating clinical benefit of nitazoxanide administration over placebo in patients with severe acute influenza [106].

Given the considerable anti-influenza efficacy and oral bioavailability of oseltamivir, many research groups derived new small molecules from its scaffold, attempting to optimize its structure to achieve higher antiviral activity and to cope with oseltamivir-resistance issue. The Chinese company ZBD Pharmaceutical developed a promising oseltamivir analogue, **HNC042**, which demonstrated potent inhibitory activity against wild-type and oseltamivir-resistant NA-H274Y viruses in preclinical studies. A phase I trial (NCT04603989) revealed good safety, tolerability, and pharmacokinetic profiles of HNC042. Also another oseltamivir derivative, **AV5080** (Viriom, Russia), resulted in vitro highly active against a wide range of influenza viruses, including oseltamivir-resistant strains. Orally administered AV5080 showed linear pharmacokinetics and good physicochemical and metabolic properties [107]. Moreover, this new NAI underwent a dose-ranging phase II clinical trial (NCT05095545), in which it showed a favourable safety and tolerability profile in patients with uncomplicated influenza, without significant side effects. Currently, this drug is undergoing a phase III clinical trial in patients with uncomplicated influenza (NCT05093998). In addition to this above-mentioned small-molecule NAIs, Cidara Therapeutics Inc. (USA) developed **CD388**, a multivalent conjugate of a dimeric NAI with a proprietary variant of a human antibody fragment engineered for prolonged half-life. This represents a novel antiviral approach able to provide in the same molecule a both protective and therapeutic effect against influenza virus infection [108]. Clinical safety and efficacy of CD388 as a prophylactic agent against influenza virus were evaluated in phase I (NCT05285137) and phase II (NCT05523089) trials, in which both intramuscular and subcutaneous administration were employed (Fig. 2). The phase II clinical trial (NCT05523089) has recently concluded and demonstrated that prophylactic administration of CD388 to healthy adults challenged with influenza virus could significantly reduce patients’ viral loads compared to placebo control.

### Host-directed drugs

For the treatment of flu, as a therapeutic strategy it is also possible to address host cell factors exploited by the virus to support its replication cycle or involved in the immune response to viral infection (Fig. 3). Thanks to this approach, several steps of the influenza virus replication cycle can be indirectly targeted and halted. The majority of these host-directed agents currently under clinical investigation are drugs originally developed for the treatment of other pathologies that have been subsequently repurposed for influenza treatment [109]. To date, the only host-targeting drug developed specifically for treating influenza is **DAS181** (or **Fludase**), a recombinant fusion protein, composed of the *Actinomyces* spp. sialidase catalytic domain and a cell surface anchored sequence. DAS181 is able to remove the sialic acid residues used by the influenza virus as receptors for its attachment and subsequent entry from the epithelial cells of the respiratory tract. In fact, this recombinant protein showed broad-spectrum antiviral efficacy both in vitro and in vivo by interfering with influenza virus entry into the host cell [110]. A phase II clinical trial (NCT01037205) demonstrated that in patients with seasonal flu continuous treatment for 3 days with 10 mg/day of DAS181 significantly decreased viral shedding compared to placebo groups [111]. However, in two Phase I studies (NCT00527865 and NCT01651494) DAS181 revealed to be not suitable for continuous treatment beyond 7 days because of the induction of anti-DAS181 IgG antibodies, causing respiratory adverse effects in treated patients [112].

**Atorvastatin**, a 3-hydroxy-3-methylglutaryl-coenzymeA (HMG-CoA) reductase inhibitor able to reduce significantly plasma cholesterol levels [113], is another repurposed drug to treat flu. Since influenza viruses induce the synthesis of large amounts of cholesterol to enrich membrane lipid rafts for viral budding, atorvastatin can impair new influenza virions assembly by interfering with lipid droplets generation [114]. Atorvastatin was recently evaluated in a phase II clinical trial (NCT02056340) in hospitalized patients with acute influenza, in which it has been shown to reduce the severity of influenza illness. A further repurposed drug against influenza virus is **diltiazem**, a calcium channel blocker approved for treatment of hypertension due to its ability to relax blood vessels. This drug emerged as a potential anti-flu compound in a screening of host-targeted agents with influenza-inhibitory activity. This study revealed that diltiazem effectively counteracted influenza virus infection both in vitro and in vivo by regulating host antiviral genes and cholesterol metabolism [115]. A phase II study (NCT03212716) started in 2017 to evaluate the efficacy of the diltiazem-oseltamivir combination for the treatment of severe influenza, but unfortunately, it was terminated before the established end because it was affected by COVID-19 pandemic. Another example of drug repurposing for anti-influenza therapy is represented by **probenecid**, a drug approved for the treatment of hyperuricemic disorders that inhibits renal tubular urate resorption by impairing cellular organic anion transporter (OAT3). This host cell protein was discovered to be involved also in IAV replication, therefore probenecid was repurposed for influenza [116]. In some studies, it emerged that coadministration of oseltamivir and probenecid could significantly reduce oseltamivir clearance, with consequent increase of its concentration in plasma. This suggested that the use of this drug combination against influenza virus infection might allow a reduction of administered dose of oseltamivir [117, 118]. However further investigation is required to deepen the pharmacokinetics and possible adverse effects of this combined therapy.

Besides targeting host proteins supporting influenza virus infection, it is also possible to address the host immune system to attenuate the antiviral immune response with the use of host-directed immunomodulators (Fig. 3). During an infection in the respiratory tract, a large number of neutrophil granulocytes are recruited and this can be associated with severe flu symptoms [119]. **Danirixin** (GSK1325756) is a small-molecule CXCR2 antagonist that in preclinical studies demonstrated the ability to reduce neutrophil activation and consequent migration to the inflammation area. The danirixin dose-dependent impairment of neutrophils activation observed in healthy patients suggested that this molecule might be used for controlling inflammation induced by viral infection, including influenza virus infection [120]. In a phase I clinical study (NCT01453478), oral danirixin proved to be generally well tolerated by patients, but its pharmacokinetics resulted to be affected by diet, age, and other factors [121]. In a phase II trial (NCT02130193), danirixin administration proved to ameliorate respiratory symptoms and general clinical conditions in patients with mild-to-moderate chronic obstructive pulmonary disease [122]. Another immunomodulator under clinical investigation for anti-flu therapy is **Celecoxib**, a diaryl-substituted pyrazole compound capable of interfering with COX-2 enzyme activity [123]. A phase III trial (NCT02108366) in patients with severe IAV infection showed that combined therapy with celecoxib and oseltamivir reduced IL-6 and IL-10 cytokines production and mortality compared to oseltamivir monotherapy, without inducing significant adverse effects [124]. A further nonsteroidal COX-2 inhibitor currently undergoing clinical evaluation as an antiviral agent against flu is **flufenamic acid**. This drug demonstrated to attenuate inflammatory response and increase the survival rate of mice lethally infected with a A(H1N1)pdm09 strain upon combination with clarithromycin and zanamivir [125]. A phase II clinical study was undertaken to compare the efficacy of triple combination of flufenamic acid, clarithromycin and oseltamivir compared to oseltamivir monotherapy (NCT03238612).

### Drug combination approaches

In the last thirty years of clinical practice, evidence of the efficacy of combined antiviral therapy against several viruses has emerged, especially to treat HIV and hepatitis C virus infection, for which this clinical approach has become the gold standard [126, 127]. As briefly mentioned above, antivirals combination has revealed to be a valid strategy for the management of influenza, since it allows to reduce single drugs dosage, consequently limiting possible adverse effects and the emergence of resistance [124, 128]. The first in vitro evaluation of the efficacy of a drugs combination against influenza virus was reported in a study published in 1968, in which a co-treatment with amantadine and interferon in chick embryo cells revealed an enhanced inhibitory effect against IAV/WSN strain replication [129]. Successively, in 1984, the first case of a triple combination of drugs against influenza virus including interferon-alpha 2, rimantadine, and ribavirin yielded improved antiviral efficacy compared to single agents and, under certain conditions, also compared to two-drug combinations [130]. Ribavirin, a guanosine analogue, was the first drug used as inhibitor of influenza virus RdRp in vitro [131], but it was never licensed for clinical treatment of influenza due to its toxicity in vivo. Thus, from the two above-mentioned and many other subsequent preclinical studies [130, 132, 133], it emerged that it is possible to achieve in vitro an enhancement of the efficacy of anti-flu treatment by combining drugs acting on different targets, thereby halting the virus replication cycle at several steps. In parallel, many studies reported that drug synergism observed in cell-based systems correlated with synergistic effect in vivo, paving the way for the application of the combination approach in the clinical treatment of flu [35, 134–136]. Therefore, several clinical trials started to assess the possible enhancing effects of combined anti-flu therapy compared to monotherapy, after a previous evaluation of possible drug-drug interactions through pharmacokinetic studies. The combinations of different direct-acting antivirals explored so far in the clinics are reported in the inset of Fig. 1. Despite the promising preclinical results, several anti-flu drugs combinations could not provide a beneficial effect in clinics comparable to that previously observed in the animal model. For instance, combined therapy with oseltamivir and peramivir did not demonstrate superiority over oseltamivir monotherapy in adults infected with IAV (H7N9) [137]. Moreover, in a phase II study (NCT01227967), a triple combination of oseltamivir, rimantadine, and ribavirin resulted in a significant decrease of viral loads versus oseltamivir monotherapy, but did not improve significantly final clinical outcomes [138]. More recently, viral RdRp-targeting drugs have been licensed, thereby offering new candidates for combined therapy against flu in the clinics (Fig. 1). In this context, a phase III clinical trial (Flagstone; NCT03684044) demonstrated that the combination of SOC NAIs with baloxavir did not provide a significant clinical benefit to hospitalized patients with severe flu compared to NAI monotherapy [139]. In another study, analysing the data of two separate prospective trials conducted in critically ill patients with flu, combined antiviral therapy with favipiravir and oseltamivir led to higher clinical improvement on day 14 in comparison to oseltamivir monotherapy [140]. As anticipated previously, despite the interruption of its developmental program, the drug pimodivir was tested in combination with oseltamivir in a phase II clinical trial (NCT02532283) in hospitalized patients with influenza. In these patients, the combined therapy resulted in a shorter time to symptoms resolution and a lower incidence of flu-related complications compared to oseltamivir monotherapy, but larger studies are required to prove clinical significance [141]. Furthermore, as described in previous paragraphs, several new anti-influenza drugs under clinical investigation, both virus- and host-directed, have been tested in clinics in combination with already approved anti-flu agents, mainly with oseltamivir [62, 96, 115, 118, 124, 125]. To date, in general, combinations of virus-directed drugs and host-directed agents yielded more positive clinical outcomes compared to single drugs administration in terms of both viral shedding reduction and resolution time of flu-related symptoms, as well as decrease of resistant variants selection. In particular, the combination of a virus-targeting drug with anti-inflammatory/immunomodulatory agents has emerged as one of the therapeutic approaches with the most promising results. For instance, the combination of oseltamivir and celecoxib, a nonsteroidal inhibitor of COX-2, is worth mentioning since in a Phase III clinical trial this combined therapeutic regimen produced a significant reduction in mortality compared to oseltamivir monotherapy [124]. In addition, the combination of oseltamivir, clarithromycin, and naproxen (a nonsteroidal anti-inflammatory drug inhibiting both COX-1/COX-2 and NP nuclear export) resulted in more rapid clinical improvement in both adult and paediatric patients infected by influenza virus compared to oseltamivir monotherapy [96, 142]. However, to better investigate the clinical efficacy of anti-flu combination therapy, further and larger clinical trials are required, including different patients’ cohorts, in particular at-risk patients who could obtain the greatest benefit from such a therapy.

## Promising anti-influenza agents under preclinical investigation

The current arsenal of approved therapeutic agents against the influenza virus is still quite limited; therefore, new antiviral drugs are needed, possibly endowed with novel mechanisms of action that could provide a higher barrier to drug resistance. For this reason, new and already explored viral and host macromolecules have been investigated as possible targets for anti-flu therapy, attempting to exploit the most conserved regions of these molecules to achieve at the same time broad-spectrum anti-influenza activity and low propensity to induce drug resistance. In the last twenty years, numerous new antiviral compounds have been developed and evaluated in preclinical studies, acting either on the virus itself or on host factors involved in viral replication or in cellular immune response (Table 2). In addition to well-established routes of administration of anti-influenza therapeutics (i.e., oral, intravenous, via inhalation, intranasal, intramuscular, and subcutaneous), in recent years some research groups have investigated novel alternative strategies of administration to improve targeted effects, bioavailability, and controlled drug release (Fig. 2). Among these, nanotechnology-based platforms have emerged, which exploit lipid/polymer-based, inorganic, or hybrid nanoparticles for targeted pulmonary delivery of the selected drug. In addition, recently hydrogel-based systems and microneedle patches have been developed as non-invasive medical devices for transdermal injection of antiviral drugs to enable sustained drug release over time [143] (Fig. 2).

### Strategies inhibiting viral entry and release

#### Targeting hemagglutinin

Hemagglutinin (HA) is the main gateway for influenza virus entry into the host cell, thus it has always been considered a crucial target for limiting viral spreading. As discussed previously, several monoclonal antibodies targeting influenza virus HA stem are already undergoing clinical evaluation, but many others are currently under preclinical investigation, directed toward either the stalk or the head of HA. The head of HA presents a higher immunogenicity compared to the stalk, leading to the production of antibodies characterized by very potent neutralizing activity but also by limited spectrum given the antigenic hypervariability of HA head across the subtypes. Nevertheless, by targeting the conserved regions of the receptor binding site (RBS), responsible for the direct binding to SA residues, it is possible to develop broadly active HA head-directed antibodies. Some examples of these cross-neutralizing mAbs have been reported, such as C05, S139/1, F045-092, S5V2-29, H2214, and Flu-20, endowed with broad-spectrum reactivity against several IAV subtypes [144–148], and mAbs CR8033 and C12G6, which cross-neutralize both IBV lineages [149, 150]. To date, no HA head-targeting mAb with cross-reactivity against both IAV and IBV has been identified, due to high sequence and glycosylation patterns dissimilarity between the two influenza virus types. On the other hand, several broadly neutralizing HA stem-directed mAbs have been developed, given the higher level of conservation of HA stalk among IAVs and IBVs. However, two distinct groups can be distinguished within IAV based on HA stalk sequence diversity: group 1, to which H1/2/5/6/8/9/11/12/13/16 belong, and group 2 to which H3/4/7/10/14/15 belong. Anti-HA stem antibodies currently under clinical investigation can be subdivided into four categories according to their cross-reactivity: group 1-specific, group 2-specific, group 1 and 2 heterosubtypic, and IAV and IBV heterosubtypic. A large number of group 1- and group 2-specific antibodies have been identified, such as CR6261 and CR8020. These mAbs target either the Trp-21 hydrophobic pocket, or the helix A region, or a membrane-proximal segment of HA stem [55, 57, 151, 152]. Although it is more difficult to obtain cross-neutralizing activity against both group 1 and group 2 HAs, some research groups succeeded in isolating broadly reactive heterosubtypic monoclonal antibodies, such as mAbs 31.a.83, 56.a.09, FI6v3, and notably MEDI8852 and MHAA4549A, currently undergoing clinical evaluation [59, 63, 153, 154]. Because of the considerable phylogenetic distance between IAV and IBV, very few antibodies with broad reactivity against both influenza types have been developed so far, e.g., mAb CR9114, which demonstrated neutralizing activity in vitro and in vivo against both influenza virus types [149]. Besides targeting directly viral HA stem or head, anti-HA antibodies can exert their antiviral effect in vivo also by mediating host immunological mechanisms to counteract viral infection via their Fc portion, such as antibody-dependent cellular cytotoxicity (ADCC), antibody-dependent cellular phagocytosis (ADCP), and complement-dependent cytotoxicity (CDC) [155, 156]. A further indirect strategy through which anti-HA antibodies can display their neutralizing activity is based on the steric inhibition of viral NA, leading to the impairment of new influenza virions release [157]. In addition to broadly neutralizing antibodies, some research efforts were also devoted to the identification of small-molecule inhibitors of viral HA, which are more suited for oral administration compared to mAbs. Similarly to previously reported antibodies, most of the anti-HA small molecules developed so far target the stalk by stabilising HA pre-fusion conformation, thus preventing viral uncoating [158–160]. In particular, two promising small-molecule inhibitors of viral fusion were developed to date, i.e., JNJ4796, identified from a screening using mAb CR6261 epitope as a reference [161], and ING-4666, a molecule derived from optimization of 4-aminopiperidines [162]. Both these inhibitors exhibited potent broad-spectrum antiviral activity in vitro and protective effects in mice against lethal influenza challenge. Moreover, ING-466 proved to synergize in vivo with both oseltamivir and baloxavir, yielding significantly increased survival rates [163]. A further category of entry inhibitors is represented by anti-HA peptides. Peptides that bind to HA head impair viral attachment to cellular receptors [164, 165], while peptides able to interfere with HA stalk conformation rearrangement prevent virus-cell fusion, similarly to Flufirvitide 3 [72, 166]. The main issue concerning these anti-HA peptides is represented by their poor stability and unfavourable pharmacokinetic properties, for which a possible solution could be the development of peptide-drug conjugates. Finally, another class of anti-HA compounds under preclinical evaluation is constituted by carbohydrate-binding agents (CBAs), molecules able to associate to the numerous glycans of HA head and stem, thereby interfering with SA residues binding and subsequent viral entry. These CBAs are mainly proteins, especially lectins, isolated in nature from a broad variety of species, including prokaryotes, algae, sea corals, fungi, plants, invertebrates, and vertebrates [167, 168]. To date, some preclinical studies uncovered the anti-influenza activity of mannose-binding lectins, the collectins surfactant protein D, the prokaryotic cyanovirin-N, the hyacinth bean lectin FRIL, and many others [169–172]. These natural agents revealed antiviral potential also against other enveloped viruses characterized by highly glycosylated surface proteins, such HIV, flaviviruses, and coronaviruses [168].

#### Targeting host factors involved in viral entry

With the purpose to limit the induction of drug resistant variants, host cell proteins involved in viral attachment/fusion and budding have also been considered as potential targets for hindering influenza virus shedding. In fact, besides sialic acid receptors, also other cellular factors have demonstrated to contribute to the efficient entry of IAV into the host cell, such as transmembrane Ca^2+^ channels, protein kinase C (PKC), phosphoinositide 3 class II β (PIK3C2β), epidermal growth factor receptor (EGFR), and substrate 8 of epidermal growth factor receptor pathway (EPS8) [173–177]. In a preclinical study conducted by a Japanese research group, it was demonstrated that IAV HA binds to cellular transmembrane voltage-dependent Ca^2+^ channels to activate Ca^2+^influx for promoting its entry phase. Based on this discovery, these scientists treated IAV-infected mice with calcium channel blockers, via intranasal inoculation, and noticed a dose-dependent decrease of viral progeny and increased survival in comparison to the untreated group [174]. PKC plays a role in endocytosis, process through which influenza virions are internalized into the host cell. A specific small-molecule inhibitor of classical PKCs, Gö6976, has been developed and exhibited the ability to impair influenza virus entry [178]. Moreover, M85, a compound targeting host kinases EGFR and PIK3C2β, showed in vitro antiviral activity against a broad variety of influenza viruses via interference with influenza virions endocytosis. In addition, it exhibited synergistic activity with oseltamivir both in vitro and in vivo, yielding a higher protective effect against lethal viral challenge compared to oseltamivir monotherapy [177]. Additionally, some host proteases are also required for viral entry, since they cleave the inactive precursor of hemagglutinin, HA0, into its two active subunits HA1 and HA2, essential for efficient viral attachment and fusion to the host cell [179]. All human influenza viruses and low pathogenic avian viruses are characterized by HAs endowed with a monobasic cleavage site that can be cleaved specifically by host epithelial transmembrane proteases like matriptases, human airway trypsin-like protease (HAT), mammalian transmembrane protease serine 2 (TMPRSS2), thus restricting the infection to human and avian respiratory tract and also to digestive system in birds. Conversely, HAs of highly pathogenic avian viruses, such as H5 and H7, possess multibasic cleavage sites that can be recognized by host proteases ubiquitously expressed in many cellular types, such as furins and convertase 5/6, and this accounts partially for the higher severity of infections by these pathogens, affecting multiple organism systems [180]. In light of their crucial role in viral entry, some inhibitors of these host proteases were tested for antiviral activity, such as the synthetic matriptase inhibitors MI-432 [181], MI-021 [182], MI-701 [183] and BAPA [184]. All these compounds exhibited inhibitory effect against influenza virus entry. Notably, combined therapy with BAPA and oseltamivir demonstrated enhanced anti-influenza activity compared to oseltamivir monotherapy in airway epithelial cells [184]. In addition, aprotinin, a bovine lungs-derived polypeptide already approved in humans as anti-protease compound for treating pancreatitis and bleeding, inhibited influenza virus replication both in vitro and in vivo through impairment of HA cleavage [185, 186]. Moreover, its administration by inhalation showed clinical effectiveness against influenza and parainfluenza viruses, leading to its approval as an anti-influenza agent in Russia as aerosol formulation [187].

#### Targeting neuraminidase

Neuraminidase (NA) is a key protein for influenza virus replication due to its role in both degrading the respiratory tract mucus to facilitate viral access to respiratory epithelium cells and in removing SA residues from HA to allow viral budding. In fact, most of the anti-flu drugs available in clinics are NAIs, designed mainly as SA analogues to inhibit NA catalytic activity. In addition, several analogues of oseltamivir and zanamivir have been synthesized and evaluated so far. Notably, there are some derivatives that were designed with the addition of particular substituents in oseltamivir scaffold to exploit the 150-cavity present next to the active site in group-1 NAs, achieving a higher antiviral potency in comparison to parental oseltamivir [188–199]. Among zanamivir analogues, its dimeric, trimeric, and tetrameric derivatives revealed antiviral activity both in vitro and in vivo superior to zanamivir [200, 201]. In addition to the design of these analogues, also new scaffolds were explored for developing novel NAIs, e.g., cyclopentane [202, 203], and pyrrolidine [204, 205]. Moreover, 2,3-difluorosialic acid and its derivatives were proposed as the first covalent inhibitors of viral NA, providing permanent inactivation of its enzyme activity [206]. Unfortunately, all of these small molecules can potentially induce the emergence of drug resistance, to which peptides are less prone. Therefore, also some peptides targeting viral neuraminidase have been developed, e.g., Peptide P2 and IntPep, which showed the ability to inhibit NA sialidase activity, consequently halting influenza virus budding [207–209].

In addition to anti-NA small molecules, monoclonal antibodies targeting viral neuraminidase were considered as potential prophylactic and therapeutic agents against influenza. Despite NA lower abundancy on viral surface compared to HA, NA-directed antibodies are produced during a natural influenza virus infection in humans and are endowed with cross-reactive neutralizing activity and protective effect [210]. Since NA-reactive antibodies have demonstrated to be an important independent correlate of protection [211–213], anti-influenza vaccination should elicit anti-NA immunity. However, unfortunately, NA antigen presents poor stability [214]. Hence, several groups are working on the development of anti-NA Abs capable of both inhibiting NA sialidase activity, by interfering sterically with NA’s access to SA receptors, and inducing Fc-mediated immune responses (ADCC, ADCP, CDC), essential for achieving heterosubtypic protection in vivo [215]*.* The main obstacle in the design of these antibodies is represented by the great antigenic diversity IAV and IBV NAs, despite the high level of conservation of the inner catalytic site. Recently, some research groups reported potent anti-NA antibodies isolated from naturally infected or vaccinated patients. IG01, derived from a patient infected with seasonal IAV (H3N2) [216], DA03E17, isolated from a patient who got infected with a IAV H1N1 subtype [217], 1G05 and 2E01, obtained from sera of IBV-infected subjects [218], and FNI9, identified from a group of healthy volunteers by the Swiss company Humabs Biomed SA [219] are the most promising. All of these NA-reactive antibodies revealed broad-spectrum potent activity against both IAVs and IBVs, conferring also high protection from influenza infection in vivo*.* Additionally, FN19 exhibited synergistic activity with HA stem-directed mAbs, suggesting a possible complementary approach for both prophylaxis and treatment of flu. Similarly to HA, viral NA also undergoes abundant glycosylation as post-translational modification. Hence, this glycoprotein can be targeted by the previously reported CBAs as well, contributing, along with HA inhibition, to impairment of influenza virus replication.

### Strategies inhibiting viral transcription and replication

#### Targeting nucleoprotein

Given its multiple crucial functions and the high conservation level among IAVs and IBVs, viral nucleoprotein (NP) has been also selected as a promising target for anti-flu therapy. Current NP inhibitors under preclinical assessment can be categorized into three classes according to their mechanism of action: (i) inhibitors of the interaction between NP and viral RNA, (ii) inhibitors of NP oligomerization, and (iii) inhibitors of NP nuclear export [220]. Among the compounds preventing the binding between NP and viral RNA, mycalamide A and KR424 emerged as promising antivirals, demonstrating also interference with vRNPs nuclear transport [221, 222]. Nucleozin is the most investigated NP oligomerization inhibitor; in fact, some analogues were derived from it with the aim of increasing the potency and spectrum of its antiviral activity [223–225]. Nucleozin acts by dysregulating aggregation of NP monomers into oligomers, causing an uncontrolled accumulation of nucleoprotein in the cellular nucleus with consequent impairment of different stages of influenza virus replication cycle [226]. In addition to the drug naproxen, currently undergoing clinical evaluation (see above), other inhibitors of NP nuclear export were developed, such as compounds ZBMD-1, KR-23502, and FA-6005 [227–229]. The two last mentioned revealed a broad antiviral effect against both IAVs and IBVs, hampering viral RNPs trafficking and subsequent assembly of newly synthesized influenza virions. FA-6005 was also found to interfere with NP-mediated nuclear import of vRNPs [229].

#### Targeting RdRp subunits

As described above, to date the only influenza virus RdRp inhibitor licensed worldwide in clinics is BXM. However, the viral RNA polymerase complex remains an interesting and promising target for anti-influenza therapy given its high level of sequence and structure conservation. Starting from BXM, several analogues were developed besides ZX-7101 (already discussed above among the compounds under clinical evaluation), some of which demonstrated in vivo similar or even superior antiviral efficacy in comparison to the parental drug [230–232]. In addition to BXM and its derivatives, many other small-molecule CENI with distinct chemical structure were identified, including flutamide derivatives [233, 234], diketonic acid derivatives [235–237], hydroxylated heterocycles [238–241], and catechol derivatives [242–244]. With regard to PB2 cap-binding domain inhibitors, several pimodivir derivatives were synthesized, some of which exhibited in vitro higher antiviral activity than the original drug [245–247]. Furthermore, some PB2 inhibitors with new chemical structure were identified, such as compounds Cap-3 and Cap-7 [248], the natural flavonoid dihydromyricetin [249], and compound PB2-39 [250]. As far as PB1 inhibitors, besides the nucleoside analogues favipiravir, molnupiravir, and ribavirin, often associated with serious side effects, also some non-nucleoside compounds were developed, such as compounds 367 [223], ASN2 [251], and 11q [252]. In particular, this last candidate drug revealed good oral bioavailability and antiviral effect against IAV in the mouse model [252].

Intersubunit interactions inhibitors represent a promising next-generation class of anti-flu agents targeting the viral RdRp [253–256]. The interfaces involved in the interactions between the influenza virus RNA polymerase subunits—PA, PB1, and PB2—are highly conserved given their pivotal role for the RdRp correct assembly and function. Thus, they represent optimal targets for direct-acting antiviral development based on the dissociation of protein–protein interactions. To this regard, small molecules or peptide inhibitors mimicking one of the regions involved in the intersubunit interaction are used to prevent protein complexes formation [257, 258]. Among the influenza virus RdRp subunit interactions, the PA/PB1 interface resulted the most suitable for inhibition by small-molecule inhibitors, since very few residues drive the subunits binding. Moreover, the PA C-terminus forms a deep groove that can accommodate better a small molecule compared to the PB1/PB2 interface, which results more flat [259–261]. Several small molecules were reported as inhibitors of RdRp PA/PB1 interaction able to interfere with PA/PB1 heterodimerization, RNA polymerase activity, and consequently viral replication [262–279]. Interestingly, Nannetti et al. evaluated the barrier to drug-resistance of some PA/PB1 dissociative compounds by passaging several times an influenza A/H1N1 virus in the presence of increasing concentrations of such compounds. From these experiments, it resulted that PA/PB1 inhibitors are less prone to reduce drug sensitivity compared to oseltamivir [273]. Similar results were obtained with structurally unrelated anti-flu compounds with the same mechanism [280]. These results support the hypothesis that emergence of resistance with dissociative inhibitors is less likely to arise, since two or more complementary mutations in both interaction partners should occur [281]. Recently, one of the most potent PA/PB1 interaction inhibitors—compound 54—was reported for the first time to be active also against highly pathogenic avian influenza viruses (HPAIVs) of H5 and H8 subytpes both in cells and *in ovo* [282]. These findings provided the proof-of-concept that compounds acting by this innovative mechanism could also be useful against viral strains of avian origin. Moreover, compound 54 acted synergistically with the NAIs oseltamivir and zanamivir both in cell cultures infected with IAV or IBV and in an embryonated egg model infected with HPAIVs, paving the way to the development of anti-flu combination therapies [282]. However, these dissociative inhibitors need further chemical optimization prior to in vivo testing because of their unfavourable physiochemical properties. In this context, a new PA/PB1 inhibitor with increased, but still suboptimal, solubility was recently reported [278], indicating that in the future dissociative PA/PB1 inhibitors might be developed for use in clinical therapy. Additionally, some PB1-derived peptides preventing PA/PB1 heterodimerization were developed as potential anti-influenza therapeutic agents [283–285], whose stability and delivery to their target site remain a major issue from a clinical perspective. With an analogous approach, some research groups worked on the design and development of novel PB1/PB2 interaction inhibitors, although the dependence on many polar interactions and the extension of the PB1/PB2 interface make it less druggable. To date, only two peptide inhibitors of the PB1/PB2 interaction were identified [286, 287]; in addition, Yuan et al*.* developed some small molecules endowed with antiviral potential and ability to prevent PB1/PB2 heterodimerization in an ELISA-based interaction assay [288, 289].

#### Targeting host factors involved in viral transcription/replication and vRNP trafficking

Although influenza viruses encode their own RdRp, responsible for both viral transcription and replication, they require cellular RNA polymerase II (RNAP-II) for starting the synthesis of viral mRNA. In fact, RNAP-II is physically involved in viral transcription through the interaction between PA C-terminus and RNAP-II phosphorylated carboxyterminal domain. This interaction allows the binding of PA N-terminal endonuclease domain to host pre-mRNAs that are cleaved into short primers for viral transcription initiation [42, 290]. Furthermore, some RNAP-II accessory factors are needed by influenza virus to accomplish its replication cycle, such as positive transcription elongation factor 1b (pTEF1b), a complex composed by cyclin-dependent kinase 9 (Cdk9) and Cyclin T1 [291]. Hence, some compounds impairing the activity of these host molecules have shown anti-influenza efficacy in vitro*,* like α-amanitin and actinomycin D, inhibiting RNAP-II [257], and 5,6-Dichloro-1-beta-d-ribo-furanosyl-benzimidazole, targeting Cdk9 [292]. For efficient trafficking of viral vRNP complexes between the host cell cytoplasm and nucleus, the involvement of some cellular proteins is also required, primarily importins and nuclear export factors [293]. Hence, the inhibition of these host cell factors also emerged as a possible strategy for impeding vRNP correct assembly and impairing new viral progeny production. Mohl et al. focused on the search for inhibitors of the interaction between viral PB1 subunit and the cellular importin-β RANBP5, which is needed for the efficient nuclear translocation of PA/PB1 heterodimer [294]. In particular, they identified a promising small molecule, compound 32, which demonstrated in vitro anti-influenza activity, ability to impair PB1 nuclear accumulation, and did not induce resistance emergence after ten passages in a immunofluorescence-based antiviral assay [294]. Another host cell protein involved in influenza virus ribonucleoprotein transport is exportin 1 (XP01), which mediates the nuclear export of viral RNPs by binding viral NEP. Based on this evidence, Perwitasari et al*.* tested verdinexor (KPT-335), a selective inhibitor of XP01 approved for treatment of lymphoma in companion dogs, against influenza virus infection, demonstrating its anti-influenza efficacy both in vitro and in vivo through the block of XPO1-mediated export of viral RNP complexes [295]. Moreover, the cellular Raf/MEK/ERK and the IKK/NF-Kb transcription pathways were demonstrated to contribute to efficient nuclear export of viral RNPs, thus emerging as new potential antiviral targets [296]. Therefore, inhibitors of these host cell pathways, like the molecule U0126 (inhibitor of MEK) and acetylsalicylic acid (targeting NF-kB), were tested against influenza virus infection both in vitro and in vivo*,* demonstrating the ability to impair the translocation of vRNPs from the nucleus to the cytoplasm, thereby hampering influenza virus propagation [297, 298].

### Strategies involving host cell antiviral immune response

#### Modulation of antiviral innate response

The innate immune system is the first host front of battle against viral infections, including influenza virus. It activates to limit the replication and spread of the invading pathogen immediately after viral entry into the organism. Notably, this system possesses many sensors, known as Pattern Recognition Receptors (PRRs) able to recognize viral Pathogen Associated Molecular Patterns (PAMPs), triggering the activation of the host antiviral state. The two main classes of host innate response sensors are RIG-I-like receptors (RLRs) and Toll-like receptors (TLRs) that are located in the cellular membrane, in the cytosol, and in the endosomes, and can detect viral genomic RNA, dsRNA, and short RNA molecules generated during viral infection (Fig. 3). The cellular pathways induced by these PRRs lead eventually to the activation of the transcription factors NF-kB and IRF-3/IRF-7, which trigger the expression of pro-inflammatory cytokines and of Type I and III interferons (IFN), respectively (Fig. 3). In turn, interferons activate the expression of hundreds of IFN-stimulated antiviral genes through binding of their specific receptors, resulting in the further production of pro-inflammatory molecules and recruitment of other cells of the innate immune system [299]. RIG-I is a cellular cytosolic sensor capable of specifically recognizing viral 5′-Triphosphate-RNA (5′ppp-RNA), consequently inducing host innate immune response [300]. Since 5′ppp-RNA itself demonstrated in vitro potential as antiviral agent against influenza virus [301–303], some research efforts have been devoted to the development of RIG-I small-molecule ligands that could act as agonists of RIG-I innate immune pathway, triggering interferon and pro-inflammatory cytokines production [304]. Moreover, some research groups worked on the design of analogues of double-stranded RNA, natural ligand of many TLRs and RLRs, that led to the identification of polyriboinosinic:polyribocytidylic acid (poly(I:C)), which has been employed mainly as endosomal TLR3 agonist to trigger host innate system [305]. This compound exhibited potent protective effect in mice challenged with influenza virus via activation of TLR3 in the respiratory tract [306]. Kagocel, a drug that demonstrated safety and efficacy against influenza in some clinical trials, was recently discovered to act as agonist of TRLs, leading to activation of IFN-I and -III responses [307]. Moreover, other small molecules capable of inducing an IFN-mediated antiviral state were identified, as compound 3, whose exact mechanism of action is still unknown [308], and the anti-PB1 compound ASN2 (mentioned above), which acts by downregulating NS1 expression [251]. Given the dependence of host innate immune response on IFN activation for efficiently counteracting influenza virus infection, treatments based on the direct administration of IFN were also tested both in vitro and in vivo*,* resulting in effective impairment of viral propagation [309, 310]. These immunomodulatory strategies that have been repurposed against influenza virus infection, together with those already tested in the clinics and previously mentioned, are depicted in Fig. 3.

#### Targeting Nonstructural protein 1

In order to circumvent the host cell surveillance system and accomplish viral replication cycle, influenza viruses have developed multiple molecular strategies [311]. The major one consists in the expression of Nonstructural protein 1 (NS1) that specifically counteracts the innate IFN response. The first discovered host-antagonizing mechanism of NS1 consists of the binding of viral dsRNA to prevent the induction of RLRs that lead to IFN expression [312]. Later, evidence emerged that NS1 also interacts with cellular ubiquitin ligases TRIM21 and Riplet to halt the activation of RIG-I that triggers IFN production [313, 314]. Furthermore, NS1 antagonizes host antiviral response through interference with CPSF30 activity, a cellular protein required for processing the 3’ end of cellular pre-mRNAs [315, 316]. Additionally, NS1 is capable of impairing NF-kB pathway by interfering with IKK, thus limiting the transcription of NF-kB target antiviral genes that mainly express proinflammatory cytokines [317]. Given its multifunctional role in counteracting antiviral innate response, NS1 has emerged as a possible viral factor to be targeted with inhibitory small molecules, thus partially restoring the host innate immune response. Epigallocatechin gallate (EGCG) was identified as an inhibitor of NS1, since it decreases viral protein levels and interferes with the interaction between NS1 and viral dsRNA, resulting in decreased viral replication [318]. Some research groups developed synthetic compounds endowed with anti-influenza activity able to bind to the NS1 hydrophobic pocket responsible for the binding of CPSF30, thus likely preventing their interaction [319–321]. Moreover, baicalin emerged as a further inhibitor of NS1 that acts by disrupting the interaction NS1-p85β, leading to decreased Akt phosphorylation. This compound could affect new viral progeny production in vitro by reducing the expression of viral NP and upregulating the expression of antiviral genes RIG-I, PKR, IFN-α, and IFN-β [322].

### Targeted protein degradation: a new frontier in the design of novel anti-influenza drugs

A new pharmaceutical technology that is currently eliciting great interest in the scientific community is targeted protein degradation (TPD), which is mainly based on the development of Proteolysis Targeting Chimeras (PROTACs). These compounds are heterobifunctional molecules formed by two ligands, connected through a linker, of which one binds specifically a protein of interest (POI) to be degraded, while the other one is in charge of recruiting a cellular E3 ubiquitin ligase, e.g., von Hippel-Lindau (VHL) or Cereblon (CRBN). Thus, once the ligand has bound the POI, the assembled ternary complex POI-PROTAC-E3 ligase triggers the ubiquitination of the POI and its consequent degradation through the ubiquitin proteasomal pathway [323, 324]. Compared to classical catalytic inhibitors, PROTACs are endowed with several advantages, such as the possibility to ensure a longer biological effect, to suppress completely all the POI’s functions at once, and to reduce the drug dosage, thus also limiting possible adverse effects [325]. The first PROTACs were conceived in 2001 by Crews and collaborators [326], but it took many years to uncover the real potential of these molecules for the treatment of cancer, immune, cardiovascular, and neurodegenerative diseases [327, 328]. More recently, this class of agents has also been considered for counteracting viral infections by targeting either viral or host proteins, since these molecules present considerable selectivity and a high genetic barrier to drug resistance [270, 329]. In particular, PROTACs against coronaviruses, flaviviruses, cytomegalovirus, hepatitis B and C viruses, HIV-1, and also against influenza virus were reported to date [330–343]. As for influenza virus, so far three of its viral proteins have been selected for TPD-based approaches, i.e., HA, NA, and PA, in view of their pivotal role in virus entry and replication. NA-directed PROTACS exploting oseltamivir as an NA-binding ligand, substituted at its amino- or carboxylate moiety with various linkers connecting it to a ligand for cellular E3 ligases, i.e., VHL or CRBN, were the first reported [343]. Through this design approach, a promising PROTAC candidate, compound 8e, was developed. This PROTAC induced in vitro dose-dependent degradation of viral NA [343]. In parallel, Li and collaborators focused on the development of HA-targeting PROTACs based on oleanolic acid, a pentacyclic triterpenoid deriving from plant metabolism, which exhibited HA binding ability, but resulted devoid of antiviral efficacy. Employing this molecule as a HA-binding ligand, this research group afforded the synthesis of compound V3, a promising PROTAC endowed with broad-spectrum in vitro anti-IAV activity, ability to degrade HA in a dose-dependent manner via ubiquitin–proteasome system, and protective effect in mice against IAV infection [341]. Moreover, a natural PROTAC targeting viral PA was identified by Zhao and collaborators in the plant endophytic fungus *Aspergillus* sp. CPCC 400735. This microbial metabolite, named APL-16–5, revealed the capacity of binding both influenza virus PA and the E3 ligase TRIM25, leading to ubiquitination and subsequent proteasomal degradation of the viral protein. This compound showed in vitro anti-IAV activity and also allowed protection from viral infection in mice [339]. Very recently, a new promising TPD-based strategy similar to PROTAC has been applied for designing novel potent antivirals that is called Hydrophobic Tagging (HyT). This approach consists in the construction of chimeric molecules composed of a POI ligand bound through a linker to a hydrophobic fragment that replaces the E3 ligase ligand present in PROTACs. This hydrophobic fragment is able to mediate POI degradation alternatively via proteasome system or autophagic lysosomal pathway. Given the multiple mechanisms of actions exploited by HyT degraders, they can lead to elimination of the target in a shorter time compared to PROTACs [344]. Taking advantage of this new technology, Ma et al., developed a series of novel acyl thiourea-based hydrophobic tagging degraders targeting viral PA, among which compound 19b emerged as a very promising compound, demonstrating potent antiviral efficacy against several influenza viruses and ability to rapidly degrade PA protein [342]. Interestingly, molecular docking studies revealed that this candidate compound bound preferably to a certain region of PA C-terminus, apparently overlapping the PB1 binding site. This suggests a further mechanism of action based on PA/PB1 interaction inhibition on which 19b may rely to exert its antiviral effect along with PA protein degradation [342].

## Concluding remarks

Influenza virus infection is commonly associated to a mild seasonal respiratory disease with low mortality rate, but the risk of an outbreak with pandemic potential, as it already occurred in the past, must not be underestimated. In fact, intensive agriculture and animal farming are contributing to the increase of the frequency of spillover events of new highly pathogenic influenza viruses from wild and domestic animals to humans. Therefore, more research efforts should be focused to extend the current arsenal of prophylactic and therapeutic options available against influenza virus infection. In this review, we reported anti-flu agents targeting either the virus itself, by targeting viral molecules involved in the influenza virus replication cycle, or the host, by interfering with the function of host factors required for efficient viral replication or by enhancing the host innate immune response. Between these two approaches, direct-acting agents should be preferred in order to limit the likeliness of off-target effects and consequent adverse reactions to which host-targeted drugs are more prone. One of the major issues that undermines the efficacy of anti-flu compounds remains drug resistance emergence, mainly due to the considerable genetic variability of this virus. To date, the most valid antiviral agents in terms of barrier to drug resistance developed against the influenza virus are PB1 nucleoside inhibitors, which indeed revealed a low probability of inducing drug-resistant variants both in vitro and in vivo*.* This is due to the high conservation of the residues of the PB1 active site involved in the recognition and binding of nucleotides for new viral RNA synthesis. Unfortunately, these molecules can result toxic because of their possible incorporation into nascent cellular RNA, thus further molecular optimization of these compounds will be needed to prevent their usage by host RNA polymerases. Another promising class of anti-flu compounds less prone to drug resistance emergence is represented by protein–protein interaction inhibitors, of which PA/PB1 interaction inhibitors are the most explored so far. In fact, like PB1 inhibitors, also these molecules exhibited in vitro a notable barrier to drug resistance given the low tolerance to mutations of the residues involved in the subunit interaction and targeted by these compounds. Moreover, the recent introduction of PROTAC technology has paved the way to a novel approach for anti-influenza drugs design, aiming at exerting a more prolonged biological effect with reduced drug dosage and limiting the emergence of drug-resistant viral strains. The identification of compounds and mAbs with broad-spectrum anti-influenza activity, acting also against viruses of avian origin, and endowed with different mechanisms of action will allow the development of effective combination strategies also for these viruses that could complement the prophylaxis with the much awaited pan-influenza vaccine.

## Data Availability

Not applicable.
